# Association of pan-immune-inflammation value and atherogenic index of plasma with chronic coronary syndrome in non-alcoholic fatty liver disease patients

**DOI:** 10.3389/fendo.2025.1650319

**Published:** 2025-08-29

**Authors:** Bing Yu, Jianqi Zhao, Wenjing Zhang, Leigang Wang, Xin Zheng, Xin Li, Zhong Yao, Yao Sun, Zhaoyu Ren, Bin Liang

**Affiliations:** ^1^ Department of Cardiology, The Second Hospital of Shanxi Medical University, Taiyuan, Shanxi, China; ^2^ Department of Cardiology, The First People’s Hospital of Jinzhong, Jinzhong, Shanxi, China

**Keywords:** non-alcoholic fatty liver disease, chronic coronary syndrome, nomogram, atherogenic index of plasma, pan-immune-inflammation value

## Abstract

**Background:**

Non-alcoholic fatty liver disease (NAFLD) is linked to a higher risk of cardiovascular disease, particularly chronic coronary syndrome (CCS). However, reliable biomarkers for early CCS risk stratification in NAFLD patients remain lacking. This study aims to assess the pan-immune-inflammation value (PIV) and atherogenic index of plasma (AIP) for CCS in NAFLD patients and to construct a practical tool for personalized risk assessment.

**Methods:**

This retrospective study included 459 NAFLD patients undergoing coronary angiography. Least absolute shrinkage and selection operator (LASSO) and multivariate logistic regression were used to discover independent risk variables for CCS. A nomogram was constructed to quantify CCS risk. Model performance was evaluated by calibration curves, concordance index, and decision curve analysis (DCA). Trend tests assessed the relationship between PIV, AIP quartiles, and CCS risk, while quantile regression analyzed their associations with coronary lesion severity (Gensini scores).

**Results:**

Eight independent variables were identified. Elevated lnPIV (OR, 2.195; 95% CI, 1.564-3.125; P< 0.001) and AIP (OR, 4.147; 95% CI, 1.770-10.095; P< 0.001) were strongly associated with CCS. The nomogram demonstrated good discrimination (C-index = 0.782) and calibration. Trend tests revealed a significant positive correlation between lnPIV/AIP quartiles and CCS risk (P for trend< 0.05). Quantile regression further indicated that lnPIV and AIP positively correlated with higher Gensini scores.

**Conclusions:**

lnPIV and AIP are independent biomarkers for CCS in NAFLD patients. The nomogram provides a valuable tool for CCS risk stratification and personalized management.

## 1 Introduction

Non-alcoholic fatty liver disease (NAFLD) has emerged as the most prevalent chronic liver condition globally, with an estimated prevalence of 32.4% and a continuing upward trend (1). NAFLD is not merely a hepatic disorder but a multisystemic disease that is associated with heightened risk of cardiovascular complications, diabetes mellitus (DM), and chronic kidney disease (2). Among the cardiovascular complications, coronary artery disease (CAD) stands out due to its considerable impact on morbidity and mortality (3). In China, the prevalence of CAD has been reported to be as high as 40.9% in NAFLD patients (4). CAD is a chronic and continuously progressive disease. Depending on the stages of disease progression, CAD is typically classified into acute coronary syndrome (ACS) and chronic coronary syndrome (CCS) (5). CCS, characterized by stable but progressive accumulation of atherosclerotic plaques, accounts for a substantial proportion of CAD cases. Despite the therapeutic strategies having progressed in recent years, the clinical burden of CCS remains high (6). According to data from the American Heart Association, CCS is predicted to affect approximately 18% of adults by 2030 (7).

Coronary atherosclerosis is widely recognized as a chronic inflammatory disease of the arterial wall (8). NAFLD contributes to systemic chronic low-grade inflammation, endothelial dysfunction, and atherogenic dyslipidemia, providing a “breeding ground” for atherosclerosis progression and thereby accelerating the development of coronary artery lesions (9, 10). Consequently, identifying reliable inflammatory and lipid-related biomarkers is essential for the early detection and risk stratification of CAD, particularly CCS, in patients with NAFLD.

Given the critical role of inflammation in atherosclerosis, a range of novel biomarkers have been increasingly proposed to enhance the assessment of coronary atherosclerosis risk (11–13). The pan-immune-inflammation value (PIV), a composite index derived from peripheral blood counts, has emerged as a prognostic biomarker in several malignancies (14). Various studies have demonstrated that PIV exhibits superior predictive potential in cardiovascular disease risk assessment compared with traditional inflammatory biomarkers (15, 16). Similarly, the atherogenic index of plasma (AIP) has been recognized as a potential biomarker for adverse cardiovascular events in CAD patients (17). However, the clinical significance of PIV and AIP in assessing CCS risk among NAFLD patients has not been thoroughly investigated. Therefore, we sought to evaluate the associations of PIV and AIP with the presence and severity of CCS in individuals with NAFLD.

## 2 Materials and methods

### 2.1 Research study overview and participants

**Figure 1:**
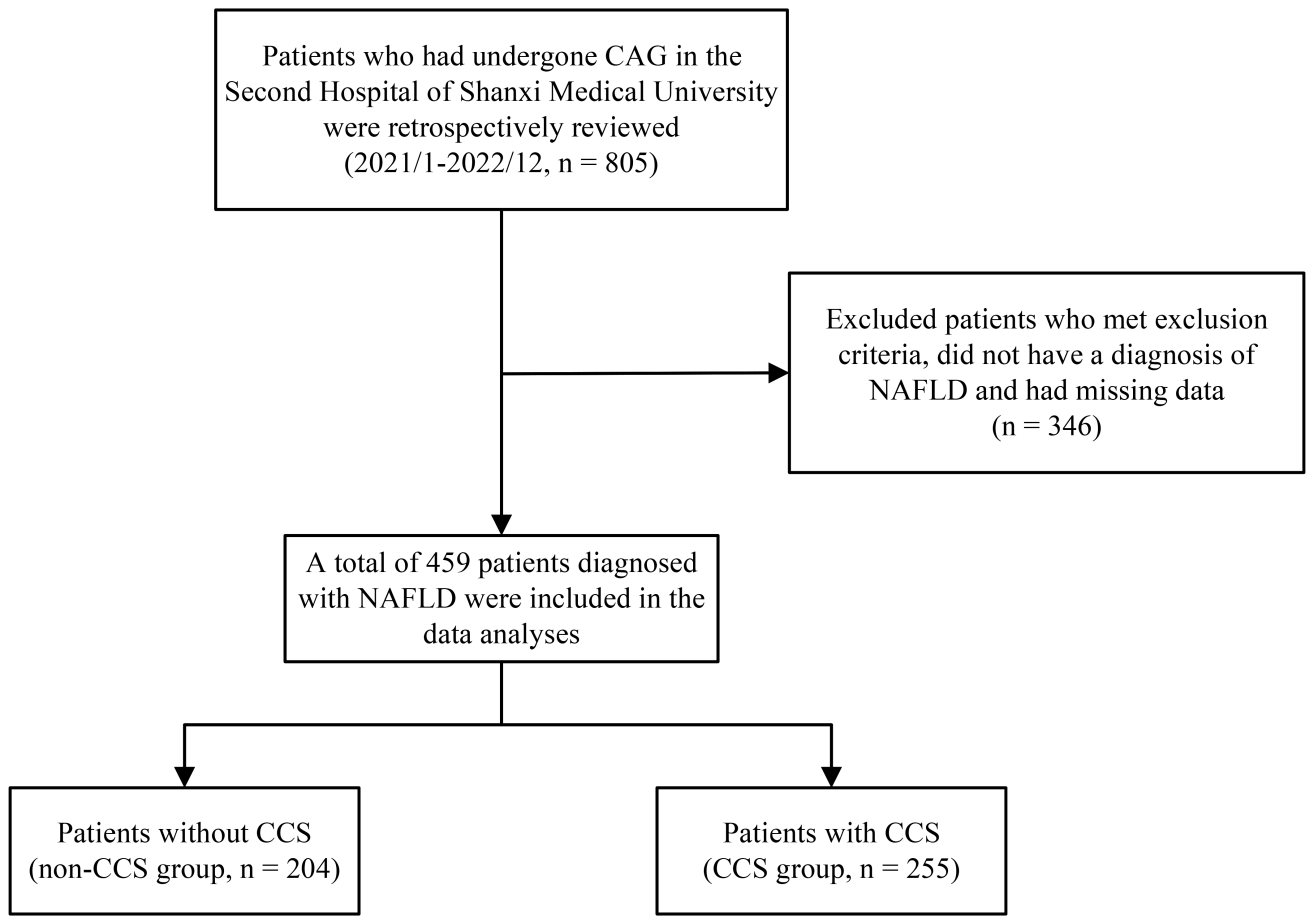
Flowchart of the subjects’ screening and grouping process.

Between January 2021 and December 2022, 805 patients who had coronary angiography (CAG) at Shanxi Medical University’s Second Hospital were included in our retrospective analysis. Following the implementation of specific inclusion and exclusion criteria, we included 459 patients with confirmed NAFLD in the final analysis (**Figure 1**). The protocol for this research project has been approved by a suitably constituted Ethics Committee of the Second Hospital of Shanxi Medical University and it conforms to the provisions of the Declaration of Helsinki. Due to the retrospective design of the study, the requirement for written informed consent was waived.

The NAFLD was diagnosed via abdominal ultrasonography following the 2017 Asia-Pacific Working Party group guidelines (18), requiring the exclusion of secondary hepatic steatosis (e.g., alcohol consumption >140 g/week for males or >70 g/week for females, viral hepatitis, or drug-induced liver injury).

Two experienced interventional cardiologists performed CAG following the Judkin’s method (5, 19). Based on the angiographic findings, the diagnosis of CCS was independently assessed, and the Gensini score was subsequently calculated to quantify the degree of coronary stenosis (**Supplementary Table 1**).

Patients were excluded if they had any of the following conditions: incomplete patient data, recent use of lipid-lowering agents, heart failure, ACS, prior coronary revascularization, structural heart disease, severe hepatic or renal dysfunction, thyroid dysfunction, hematologic or autoimmune diseases, malignancies, familial hypercholesterolemia, or systemic infections.

### 2.2 Clinical information and lab measurements

Baseline clinical and demographic data were retrospectively obtained from electronic medical records, including gender, age, body mass index (BMI), smoking history, hypertension, and DM. All data were collected at the time of admission. Fasting venous blood samples were drawn in the morning following admission, after at least 12 hours of fasting, and prior to undergoing CAG. Laboratory parameters assessed include red blood cell count (RBC), hemoglobin concentration (HGB), red cell distribution width-coefficient of variation (RDW-CV), platelet count, lymphocyte count, monocyte count, neutrophil count, aspartate aminotransferase (AST), alanine aminotransferase (ALT), total bilirubin (TBIL), serum albumin (ALB), serum creatinine (SCr), blood uric acid (URIC), blood urea nitrogen (UREA), fasting blood glucose (FBG), fibrinogen (FIB), D-dimer (D-Di), triglycerides (TG), total cholesterol (TC), low-density lipoprotein cholesterol (LDL-C) and high-density lipoprotein cholesterol (HDL-C. The platelet-to-lymphocyte ratio (PLR), monocyte-to-lymphocyte ratio (MLR), neutrophil-to-lymphocyte ratio (NLR), systemic inflammation response index (SIRI), systemic immune-inflammation index (SII), pan-immune-inflammation value (PIV), atherogenic index (AI), remnant cholesterol (RC), and atherogenic index of plasma (AIP) were calculated using the following formulas:


$$PLR=\frac{\text{platelet count}}{\text{lymphocyte count}}$$

$$MLR=\frac{\text{monocyte count}}{\text{lymphocyte count}}$$

$$NLR=\frac{\text{neutrophil count}}{\text{lymphocyte count}}$$

$$SIRI=\frac{\text{monocyte count}\times \text{neutrophil count}}{\text{lymphocyte count}}$$

$$SII=\frac{\text{platelet count}\times \text{neutrophil count}}{\text{lymphocyte count}}$$

$$PIV=\frac{\text{neutrophil count}\times \text{platelet count}\times \text{monocyte count}}{\text{lymphocyte count}}$$

$$AI=\frac{TC-HDL-C}{HDL-C}$$

RC=TC−(HDL−C+LDL−C)

$$AIP=\log_{10}\left(\frac{TG}{HDL-C}\right)$$

### 2.3 Statistical analysis

Continuous variables were assessed for normality using the Shapiro-Wilk test. Data with normal distribution were presented as mean ± standard deviation ( $\bar{\text{x}}$ ± s), and compared using the Student’s t-test. Non-normally distributed variables were presented as median (Q1, Q3) and analyzed using the Mann-Whitney U test. Count data were expressed as frequencies (%) and compared by the chi-square test. Natural logarithmic transformation was done for PLR, MLR, NLR, SIRI, SII, and PIV to minimize skewness and stabilize variance.

Initially, variable selection was screened using the least absolute shrinkage and selection operator (LASSO) regression with 10-fold cross-validation to prevent overfitting. Next, we used multivariate logistic regression analysis to identify independent variables more closely. A nomogram was constructed using significant variables from the final logistic model. The model’s discrimination was assessed using the concordance index (C-index) and receiver operating characteristic (ROC) curves. Moreover, to verify the model’s robustness, ROC curves were generated by the bootstrap method (resampling 1,000 times). Calibration of the model was assessed via calibration plots based on the bootstrap method (resampling 1,000 times) to examine the agreement between predicted and observed outcomes. The clinical utility was evaluated using Decision Curve Analysis (DCA), and the net benefit at different thresholds was quantified using the Clinical Impact Curve (CIC). Additionally, quantile regression assessed the relationships between lnPIV and AIP levels and the 25th, 50th, and 75th percentiles of Gensini scores.

Analyses were conducted using R version 4.4.1. We used two-tailed P values, and a P value less than 0.05 was considered statistically significant.

## 3 Results

### 3.1 Comparison of baseline clinical characteristics and laboratory test parameters between the non-CCS and CCS groups

**Table 1** summarizes the patients’ baseline characteristics and laboratory test parameters. The CCS group had a much greater proportion of males (64.3% vs. 52.0%; P = 0.010) and was older on average (59 [52, 66] vs. 56.5 [50.5, 63], P = 0.004) compared to the non-CCS group. Hypertension, DM, and smoking history were also more prevalent in the CCS group (61.6% vs. 46.1%; 35.7% vs. 13.7%; 50.2% vs. 31.4%; P = 0.001, P< 0.001, and P< 0.001, respectively). Additionally, laboratory findings showed levels of lnNLR, lnMLR, lnSII, lnSIRI, lnPIV, fasting glucose, TC, TG, LDL-C, AIP, and RC were significantly elevated in the CCS group (P< 0.05). In contrast, HDL-C level was lower in the CCS group (P = 0.006).

**Table 1:** Baseline characteristics of non-CCS and CCS groups.

Characteristics	Non-CCS (N=204)	CCS (N=255)	*P*
Gender (Male)	106 (52.0%)	164 (64.3%)	.010*
Age	56.50 (50.50, 63.00)	59.00 (52.00, 66.00)	.004*
BMI (kg/m^2^)	25.71 (24.22, 27.71)	26.12 (24.56, 28.01)	.146
Hypertension	94 (46.1%)	157 (61.6%)	.001*
DM	28 (13.7%)	91 (35.7%)	<.001*
Smoking	64 (31.4%)	128 (50.2%)	<.001*
RBC, 10^9^/L	4.69 (4.35, 5.01)	4.71 (4.38, 4.98)	.727
Hemoglobin, g/dL	144.99 ± 14.39	145.23 ± 15.31	.863
RDW-CV (%)	12.45 (12.00, 12.90)	12.50 (12.10, 12.90)	.165
Platelet (10^9^/L)	218.00 (184.50, 249.00)	221.00 (185.00, 253.00)	.347
Lymphocyte (10^9^/L)	1.90 (1.51, 2.28)	1.91 (1.52, 2.38)	.673
Monocyte (10^9^/L)	0.42 (0.34, 0.51)	0.48 (0.38, 0.58)	<.001*
Neutrophil (10^9^/L)	3.43 (2.73, 4.63)	4.13 (3.21, 5.44)	<.001*
PLR	115.43 (91.40, 138.01)	116.26 (93.38, 142.98)	.566
MLR	0.22 (0.18, 0.27)	0.24 (0.19, 0.30)	.001*
NLR	1.89 (1.34, 2.56)	2.09 (1.59, 3.00)	<.001*
SIRI	0.77 (0.51, 1.12)	1.00 (0.66, 1.57)	<.001*
SII	402.25 (276.21, 558.34)	481.34 (338.27, 658.17)	<.001*
PIV	156.91 (110.75, 245.58)	236.20 (141.52, 326.81)	<.001*
lnPLR	4.73 ± 0.32	4.75 ± 0.35	.485
lnMLR	-1.51 ± 0.34	-1.39 ± 0.39	.001*
lnNLR	0.64 ± 0.43	0.79 ± 0.49	<.001*
lnSIRI	-0.25 ± 0.54	0.03 ± 0.63	<.001*
lnSII	5.99 ± 0.47	6.18 ± 0.52	<.001*
lnPIV	5.10 ± 0.58	5.42 ± 0.67	<.001*
ALT (U/L)	22.30 (16.00, 32.30)	22.80 (16.80, 33.90)	.478
AST (U/L)	21.95 (18.10, 25.95)	22.20 (17.50, 27.90)	.686
TBIL (umol/L)	14.15 (11.10, 17.85)	13.50 (10.70, 16.90)	.078
ALB (g/L)	41.70 (39.80, 43.55)	40.30 (38.70, 42.85)	<.001*
Urea nitrogen (mmol/L)	5.30 (4.40, 6.10)	5.30 (4.50, 6.40)	.398
Uric acid (umol/L)	362.55 ± 83.92	358.50 ± 81.42	.601
SCr (umol/L)	65.00 (56.00, 75.00)	66.00 (57.00, 74.50)	.322
TC (mmol/L)	4.29 (3.64, 5.04)	4.53 (3.91, 5.31)	.007*
TG (mmol/L)	1.67 (1.23, 2.22)	1.81 (1.35, 2.67)	.002*
HDL-C(mmol/L)	1.14 (0.97, 1.35)	1.08 (0.93, 1.28)	.006*
LDL-C(mmol/L)	2.27 (1.81, 2.66)	2.42 (2.04, 2.84)	.004*
AI	1.39 (1.27, 1.49)	1.40 (1.32, 1.50)	.176
RC	0.85 (0.59, 1.21)	0.95 (0.74, 1.30)	.001*
AIP	0.15 (0.00, 0.31)	0.25 (0.07, 0.41)	<.001*
FBG (mmol/L)	5.29 (4.94, 5.79)	5.56 (4.90, 6.99)	.008*
D-Dimer (ng/mL)	71.00 (49.50, 112.00)	79.00 (53.00, 128.00)	.055
Fibrinogen (g/L)	2.73 (2.35, 3.19)	2.85 (2.51, 3.21)	.051

Data are expressed as mean ± standard deviation, median (Q1, Q3), or n (%). lnPLR, lnMLR, lnNLR, lnSIRI, lnSII, and lnPIV are the natural logarithms of PLR, MLR, NLR, SIRI, SII, and PIV, respectively. *P value< 0.05.

### 3.2 LASSO regression analysis for characteristics screening

**Figure 2:**
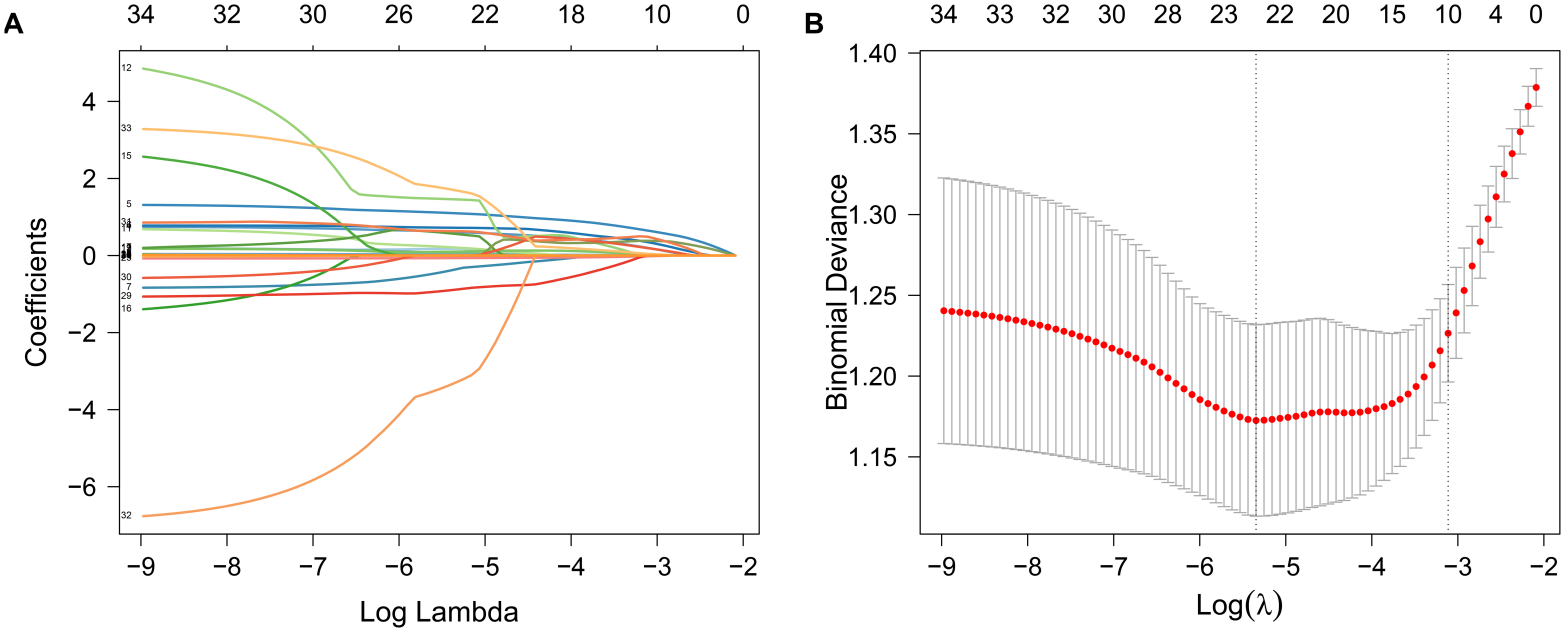
Variable selection was performed using LASSO regression analysis in combination with 10-fold cross-validation. **(A)** Path plot of LASSO regression coefficients for the independent variables constructed from log(λ); **(B)** Cross-validation error plot was used to determine the optimal penalty parameter λ. The vertical left dashed line represents λ (lambda.min) at minimum bias, while the right dashed line represents λ (lambda.1se) with one standard error to the right of lambda.min.

The main variables of CCS were initially screened using LASSO regression analysis in combination with 10-fold cross-validation (**Figure 2**), and lambda.1se was selected as the optimal penalty coefficient. Nine non-zero coefficient variables were identified: age, hypertension, DM, smoking, neutrophil count, lnPIV, ALB, LDL-C, AIP, and RC. After testing the variance inflation factor (VIF) (**Supplementary Table 2**), we retained the lnPIV and excluded neutrophil count based on the principle of minimizing redundancy and enhancing model stability.

### 3.3 Multivariable logistic regression for characteristics selection

The variables preliminarily selected by LASSO regression were further analyzed by multivariate logistic regression (**Table 2**). Among these, the variable RC was initially included in Model I but was subsequently excluded from Model II due to its non-significance (P = 0.557). The multivariable logistic regression analysis results found that age (OR, 1.035; 95% CI, 1.010-1.061; P = 0.007), hypertension (OR, 1.869; 95% CI, 1.197-2.934; P = 0.006), DM (OR, 3.149; 95% CI, 1.876-5.414; P< 0.001), smoking (OR, 2.411; 95% CI, 1.526-3.849; P< 0.001), LDL-C (OR, 1.899; 95% CI, 1.357-2.698; P< 0.001), lnPIV (OR, 2.195; 95% CI, 1.564-3.125; P< 0.001), and AIP (OR, 4.147; 95% CI, 1.770-10.095; P = 0.001) were independent risk factors for CCS in patients with NAFLD. Conversely, ALB (OR, 0.923; 95% CI, 0.870-0.975; P = 0.005) was identified as an independent protective factor. We mapped the forest plot based on these independently correlated characteristics (**Supplementary Figure 1**).

**Table 2:** Multivariate logistic analyses of variables associated with CCS in NAFLD.

Characteristics	Model I				Model II			
	OR	95%CI		P	OR	95%CI		*P*
Age	1.034	1.009	1.060	0.009*	1.035	1.010	1.061	0.007*
Hypertension	1.871	1.199	2.938	0.006*	1.869	1.197	2.934	0.006*
DM	3.178	1.891	5.473	<0.001*	3.149	1.876	5.414	<0.001*
Smoking	2.425	1.535	3.873	<0.001*	2.411	1.526	3.849	<0.001*
ALB	0.924	0.870	0.976	0.006*	0.923	0.870	0.975	0.005*
LDL-C	1.813	1.254	2.66	0.002*	1.899	1.357	2.698	<0.001*
lnPIV	2.215	1.575	3.159	<0.001*	2.195	1.564	3.125	<0.001*
AIP	3.539	1.302	9.946	0.014*	4.141	1.770	10.095	0.001*
RC	1.173	0.685	2.006	0.557				

Model I was adjusted for variables screened by LASSO regression with 10-fold cross-validation; Model II adjusted for variables with a *P* value of less than 0.05 in Model I. *P value< 0.05.

### 3.4 Nomogram construction and validation

**Figure 3:**
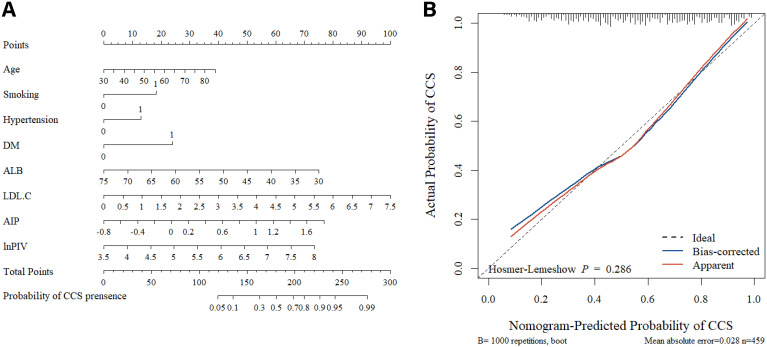
The nomogram and calibration curves. **(A)** Using the nomogram, each variable’s location on its axis is identified to assign corresponding points. These points are then summed across all predictor variables to generate a total points score. Finally, the estimated probability of CCS occurrence is determined by referencing the bottom scale. **(B)** The nomogram calibration curves demonstrated the concordance between predicted and observed probabilities. The Hosmer-Lemeshow test yielded *P* > 0.05, indicating a good model fit.

Multivariate logistic regression analyses revealed statistically significant independent variables. Based on these variables, a nomogram for CCS risk estimation in the NAFLD population was constructed (**Figure 3A**). The nomogram model’s internal validation was performed using bootstrap (resampling = 1000), and the calibration curves demonstrated a strong match between the predicted and actual probabilities of CCS (**Figure 3B**). Furthermore, the nomogram’s C-index was 0.782 (95% CI, 0.741-0.824), indicating high accuracy in predicting CCS risk.

### 3.5 Evaluation of the nomogram model’s clinical utility

**Figure 4:**
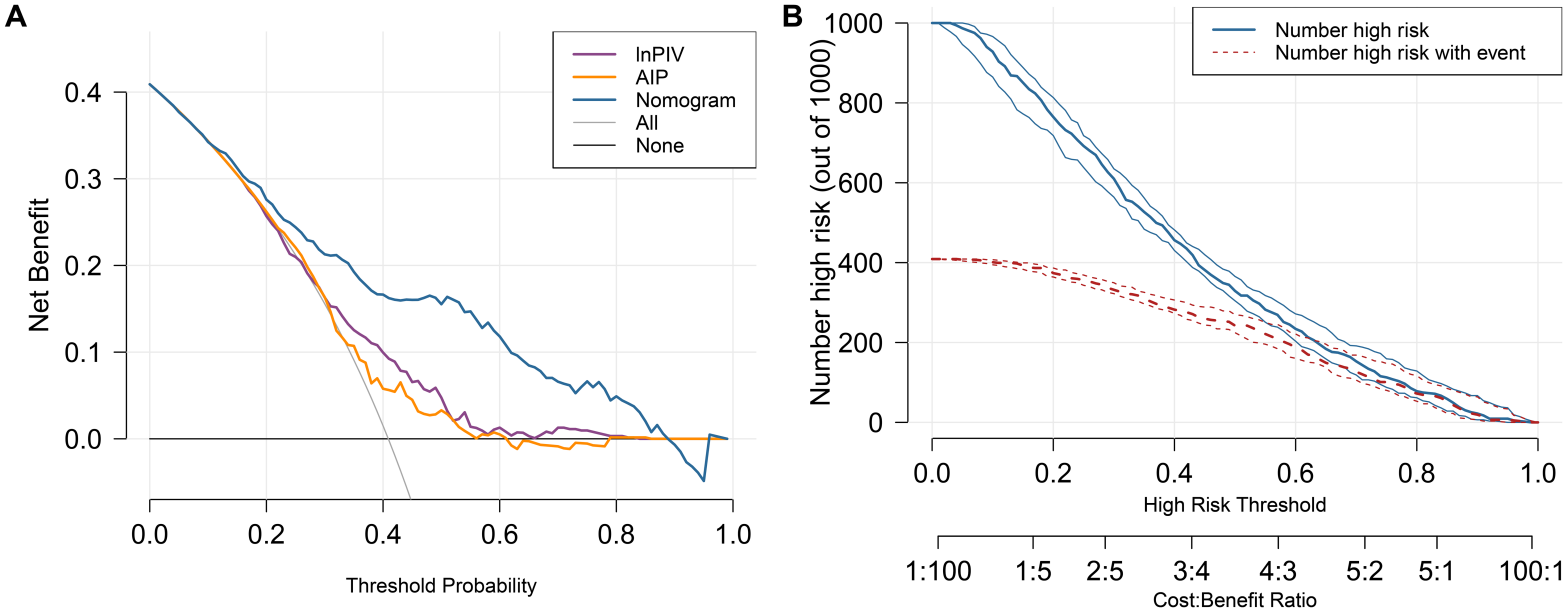
The DCA curves and CIC for the nomogram model. **(A)** DCA curves of lnPIV, AIP, and the nomogram. **(B)**The nomogram model’s CIC displays two curves: the red curve represents the actual count of positives at each threshold, while the blue curve reflects the count of individuals identified as positive by the model at each threshold.

The DCA curves (**Figure 4A**) show that the nomogram model provides higher net benefits compared to lnPIV or AIP alone and outperforms both the “no intervention” and “intervention for all” strategies across a threshold probability range of approximately 0.1-0.9. Meanwhile, the CIC (**Figure 4B**) demonstrates the correspondence between predicted and actual case numbers at different threshold probabilities. It reveals that the number of positive cases predicted by the nomogram model gradually approaches the number of actual positive cases as the risk threshold increases, indicating that the model has good predictive ability and clinical applicability.

### 3.6 ROC analysis of biomarkers and nomogram model

**Figure 5:**
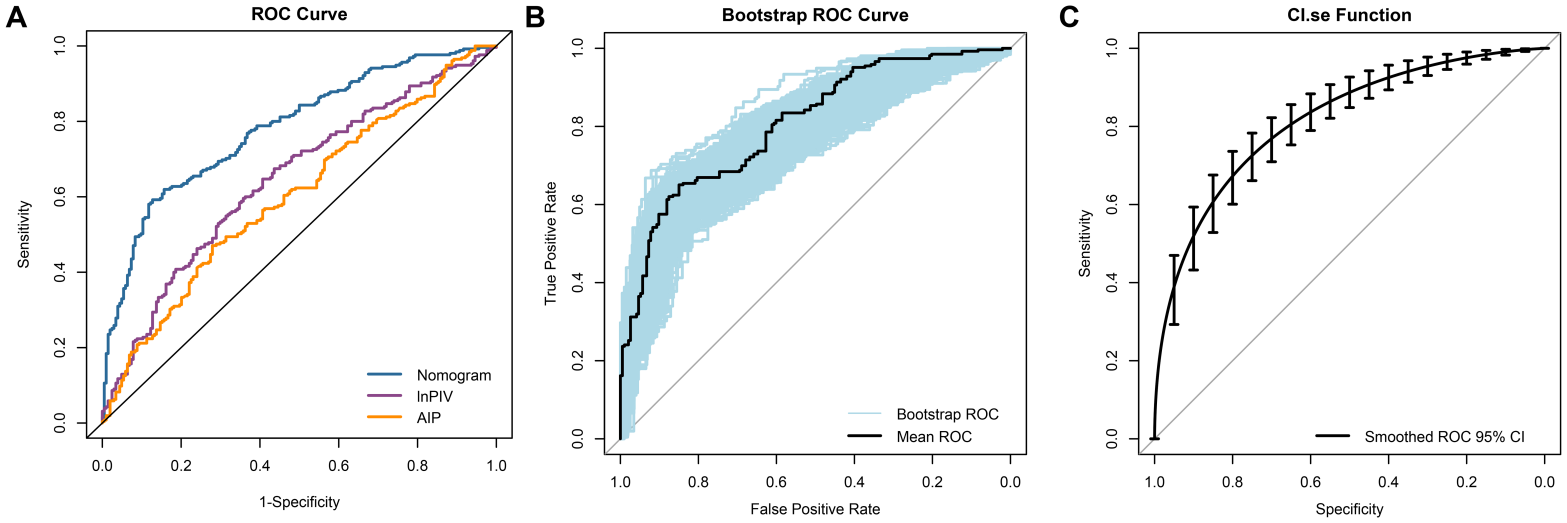
ROC curves analysis of the ability to predict CAD in NAFLD. **(A)** The ROC curves and AUC values for the nomogram model, lnPIV, and AIP are as follows: nomogram model: 0.782 (95% CI, 0.741 - 0.824); lnPIV: 0.646 (95% CI, 0.595 - 0.696); AIP: 0.602 (95% CI, 0.550 - 0.653); **(B)** The mean ROC curve displays the AUC from internal validation with the bootstrap method (resampling = 1000), yielding an AUC of 0.781 (95% CI, 0.735 - 0.823); **(C)** The dotted vertical lines indicate the 95% CI for the smoothed ROC curve, with a smoothed ROC AUC of 0.815 (95% CI, 0.775 - 0.850).

To evaluate the predictive value of each biomarker, their AUC values were calculated respectively. The results indicated that lnPIV had the highest AUC of 0.646 (95% CI, 0.595-0.696, P< 0.001), outperforming lnNLR (AUC = 0.590, 95% CI, 0.538-0.642, P = 0.001), lnPLR (AUC = 0.516, 95% CI, 0.463-0.596, P = 0.565), lnMLR (AUC = 0.587, 95% CI, 0.535-0.639, P = 0.001), lnSII (AUC = 0.607, 95% CI, 0.555-0.658, P< 0.001), and lnSIRI (AUC = 0.629, 95% CI, 0.578-0.679, P< 0.001). Additionally, the AUC of AIP (0.602, 95% CI, 0.550-0.653, P< 0.001) was significantly higher compared to AI (AUC = 0.537, 95% CI, 0.483-0.590, P = 0.176) and RC (AUC = 0.588, 95% CI, 0.536-0.640, P = 0.001). The ROC curves revealed that the AUC of the nomogram model was 0.782 (95% CI, 0.741-0.824, **Figure 5A**), which provided better discriminatory power than a single variable. After internal validation by the bootstrap method (resampling = 1000), the AUC of the mean ROC curve was 0.781 ± 0.022 (**Figure 5B**). Moreover, we fitted a smoothed ROC curve using the resampled data, estimated 95% confidence intervals for sensitivity, and presented them as error bars on the ROC plot. The smoothed ROC curve’s AUC was 0.815 (95% CI, 0.775 - 0.850) (**Figure 5C**).

### 3.7 Association of lnPIV and AIP quartiles with CCS risk in NAFLD patients

To investigate the relationship between the levels of lnPIV, AIP, and the risk of CCS in NAFLD patients, we regrouped them according to quartiles of lnPIV or AIP and analyzed them separately using trend tests (**Table 3**). Specifically, the ORs of lnPIV increased gradually across quartiles, indicating a significant positive trend (Multivariate model, P for trend< 0.001). AIP showed a similar trend, significantly associated with increased risk of CCS across quartiles (Multivariate model, P for trend = 0.004).

**Table 3:** Logistic analysis of CCS in NAFLD by lnPIV, AIP, and Nomogram points quartile.

Variable		CCS	Non-CCS	Non-adjusted		Multivariate model	
				OR (95%CI)	P	OR (95%CI)	*P*
lnPIV							
Q1	<4.802	46	69	Ref.		Ref.	
Q2	4.802-5.254	56	58	1.448 (0.859,2.452)	0.166	1.066 (0.590,1.924)	0.831
Q3	5.254-5.689	68	47	2.170 (1.286,3.694)	0.004*	2.044 (1.140,3.699)	0.017*
Q4	≥5.689	85	30	4.250 (2.451,7.514)	<0.001*	3.584 (1.944, 6.738)	<0.001*
*P* for trend				<0.001*		<0.001*	
AIP							
Q1	<0.0428	52	63	Ref.		Ref.	
Q2	0.0428-0.2023	58	56	1.255 (0.747,2.113)	0.392	1.320 (0.731, 2.393)	0.359
Q3	0.2023-0.3709	67	48	1.691 (1.006,2.860)	0.048*	1.580 (0.876, 2.864)	0.130
Q4	≥0.3709	78	37	2.554 (1.501,4.397)	0.001*	2.490 (1.346, 4.673)	0.004*
*P* for trend				<0.001*		0.004*	

Trend tests are based on the variable with a median value for each quintile. The multivariate model was adjusted for age, hypertension, smoking, DM, albumin, LDL-C, and lnPIV or AIP. *P value< 0.05.

### 3.8 Distributional effects of lnPIV and AIP on Gensini scores observed through quantile regression

**Figure 6:**
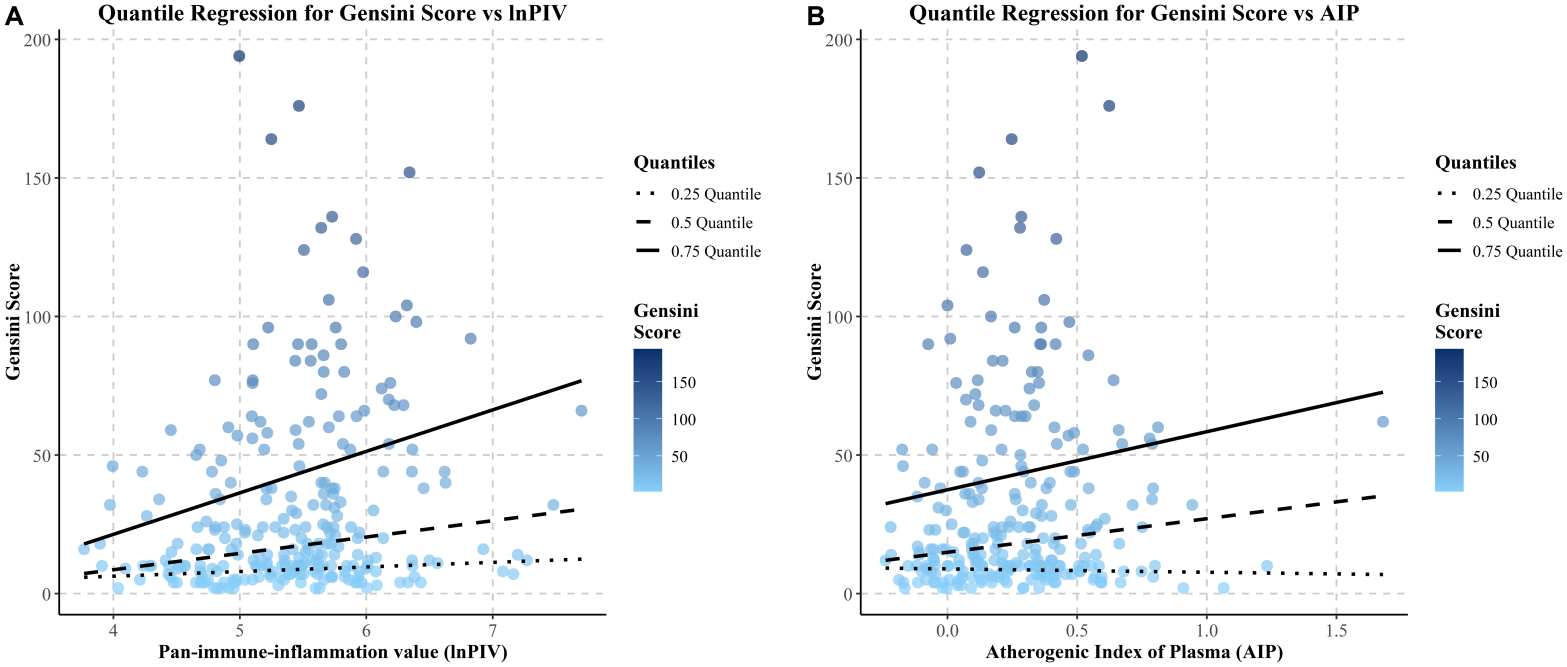
Quantile regression lines of gensini score on lnPIV and AIP across quantiles.

Quantile regression analysis assessed the effects of lnPIV and AIP at different percentiles of Gensini scores and their statistical significance (**Figure 6**). The results indicated that the regression coefficients of lnPIV were 5.90 (P = 0.045) at the 50^th^ percentile and 14.97 (P = 0.017) at the 75^th^ percentile. In contrast, the coefficient at the 25^th^ percentile was 1.661 (P = 0.153), which was not statistically significant (**Supplementary Table 3**). These findings suggest that lnPIV has a more pronounced positive effect on patients with higher Gensini scores (≥50^th^ percentile), which indicates worse coronary stenosis. Similarly, the regression coefficient of AIP at the 75^th^ tertile was 20.97 (P = 0.017) (**Supplementary Table 3**), highlighting its significant predictive value in patients with higher Gensini scores.

## 4 Discussion

In our study, we systematically assessed the combined predictive value of lnPIV and AIP for CCS in patients with NAFLD. Both lnPIV and AIP demonstrated independent associations with CCS risk and exerted a greater influence in moderate-to-severe coronary atherosclerosis, as reflected by Gensini scores. These findings suggest that systemic immune-inflammation burden and atherogenic dyslipidemia contribute to the progression of CCS in NAFLD-related cardiovascular disease. Additionally, the nomogram integrating these biomarkers exhibited robust discriminatory ability and calibration, providing a practical tool for CCS risk stratification in clinical settings.

CCS refers to a series of clinical manifestations caused by structural and/or functional abnormalities in the coronary arteries and/or microcirculation, excluding acute coronary thrombosis as the predominant cause (6). Its pathogenesis is primarily driven by maladaptive inflammatory responses and dysregulated lipid metabolism (20). NAFLD, as a metabolic disease, can induce immune signaling disturbances and maintain the body in a persistent low-grade inflammatory state (21). Besides the hepatic fat accumulation-induced inflammatory response, the enrichment of myeloid derived suppressor cells (MDSC) and natural killer T cells (NKT) in the spleen has been shown to exacerbate the hepatic inflammatory response (22). This spleen-hepatic crosstalk aggravates the systemic inflammatory response and is a key feature of NAFLD (23, 24). These inflammatory mechanisms likely serve as critical intermediaries linking NAFLD to CCS. Previous studies have shown that the atherosclerosis progression involves complex regulation of cytokines and immune cells across all stages (25). Consistent with this, our findings revealed significantly higher levels of neutrophils, monocytes, and platelets in NAFLD patients with CCS compared to those without. Monocytes are the earliest immune cells recruited to sites of endothelial dysfunction. They secrete pro-inflammatory cytokines and reactive oxygen species (ROS), differentiate into macrophages, and contribute to early atherosclerotic lesion formation by uptaking lipoproteins and becoming foam cells that secrete additional inflammatory mediators (26). Neutrophils aggravate vascular injury by secreting ROS and pro-inflammatory molecules, which in turn recruit additional immune cells and amplify inflammatory cascades (27). Moreover, lymphocytes are also actively involved in various stages of atherosclerosis. In general, T cells promote disease progression by regulating cellular interactions and releasing inflammatory cytokines, whereas B cells may exert protective effects by dampening inflammation (28). Platelets, although anucleated, can secrete many chemokines upon activation, initiating and sustaining local inflammatory processes at the site of vascular injury (29). These cellular and molecular events drive the chronic inflammatory course of atherosclerosis together, ultimately leading to the pathologic progression of CCS. The PIV, which integrates neutrophils, monocytes, lymphocytes, and platelets, serves as a composite indicator of systemic inflammation (16). In our study, the ROC curves revealed that the PIV had the highest AUC value, indicating its predictive strength and clinical relevance.

In parallel, AIP reflects the atherogenic potential of lipid metabolism and is calculated by the logarithm of the TG/HDL-C ratio. The liver plays a central role in lipid homeostasis, but NAFLD-related hepatic disorder leads to elevated TG levels, reduced HDL-C levels, and increased production of small dense LDL particles (sdLDL), which are highly atherogenic (30–32). Despite adequate control of LDL levels in some patients, a “residual risk” of cardiovascular events may still exist, which may be attributed to elevated TG and reduced HDL-C levels—key components captured by AIP (33, 34). Elevated TG is metabolized into triglyceride-rich lipoproteins (TRLs), and small, dense, low-density lipoprotein (sdLDL) particles are formed (32). TRLs deposit cholesterol in the arterial wall and mediate foam cell formation, while oxidized sdLDL further triggers an immune response and vascular inflammation (35). Conversely, HDL confers cardiovascular protection by mediating reverse cholesterol transport, reducing oxidative stress, and preserving endothelial function (36, 37). AIP has been considered more effective than individual lipid indices in predicting cardiovascular disease risk and has shown significant potential for prognosis prediction and diagnosis (38, 39). Our study revealed that elevated AIP levels significantly increased the risk of CCS in NAFLD patients, even after adjusting for traditional confounders, providing new evidence for the clinical application of AIP as a CCS risk assessment biomarker.

In addition to inflammation and lipid metabolism, ALB also emerged as an independent predictor of CCS in this study. ALB is the most abundant protein in plasma, responsible for preserving colloid osmolarity and exerting anti-inflammatory and antioxidant effects (40, 41). Our study found that among NAFLD patients, lower ALB levels were significantly linked to higher CCS risk. Although ALB levels may not decrease significantly in early NAFLD, structural alterations may impair its physiological activity (42). As the disease progresses, reduced ALB levels may further weaken the body’s antioxidant and anti-inflammatory defenses, thus exacerbating the risk of CCS.

To further confirm the link between lnPIV, AIP levels, and coronary artery severity lesions, we applied quantile regression analysis. The results indicated significance for lnPIV at the 50^th^ and 75^th^ percentiles, but significance was noted only for AIP at the 75^th^ percentile. These findings confirmed the potential of lnPIV and AIP in predicting the severity of coronary atherosclerosis, aligning with earlier research findings (43, 44), and may serve as valuable indicators for identifying individuals at greater cardiovascular risk.

While traditional cardiovascular risk factors remain essential for risk assessment, our results highlight the added value of composite indices such as lnPIV and AIP in refining the prediction of CCS, particularly among NAFLD patients. The nomogram constructed based on these biomarkers showed strong predictive accuracy and calibration, and may offer a novel, clinically applicable tool for individualized CCS risk stratification.

Several limitations should be acknowledged in this study. First, the retrospective, single-center design may introduce potential selection bias and limit the generalizability of the findings. Second, a small sample size may reduce statistical power, affecting the precision of some estimates. Third, residual confounding from unmeasured variables may influence the observed associations. Therefore, future studies should address these limitations through large-scale, multi-center cohorts and longitudinal designs to better understand the causal relationship between biomarkers and CCS. Monitoring dynamic changes in these biomarkers over time would also provide valuable insights into their role in disease progression and risk stratification.

## 5 Conclusions

Elevated PIV and AIP levels were found to be independent risk factors for CCS in NAFLD patients, showing significant associations between their quartiles and the severity of coronary lesions (Gensini scores). The nomogram developed in this study offers a valuable predictive tool, enhancing the identification of high-risk individuals. These findings have important implications for risk stratification and the development of management strategies for CCS in NAFLD patients.

## Data Availability

The raw data supporting the conclusions of this article will be made available by the authors, without undue reservation.
