# Supplementary material for: Association of pan-immune-inflammation value and atherogenic index of plasma with chronic coronary syndrome in non-alcoholic fatty liver disease patients
Source: Front Endocrinol (Lausanne). 2025 Aug 29;16:1650319. doi: 10.3389/fendo.2025.1650319 (PMC12425755; doi:10.3389/fendo.2025.1650319)
Supplement: Supplementary file 1 [file DataSheet1.docx]

Supplementary Material

# Supplementary Figures and Tables

## Supplementary Figures


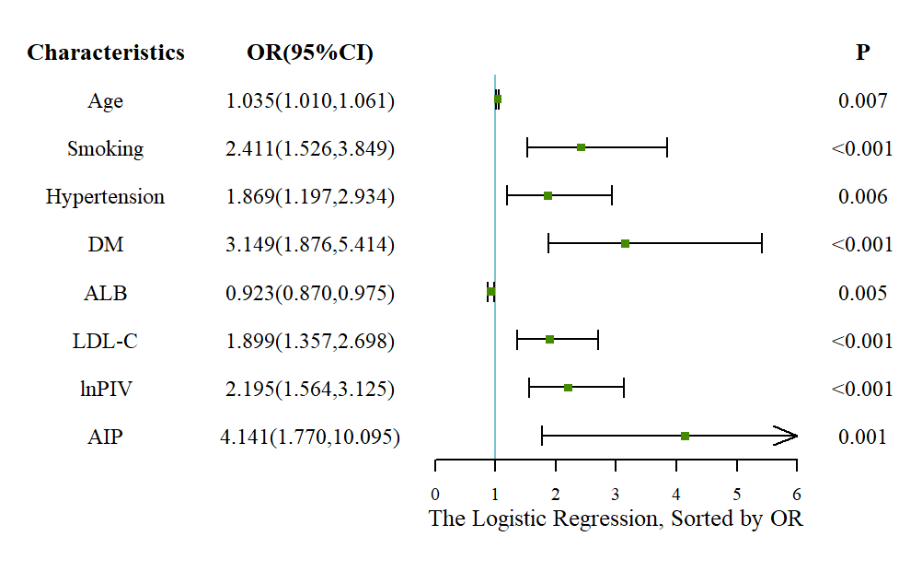


**Supplementary Figure 1.** Forest plot of independently associated factors with CCS in NAFLD

## Supplementary Tables

**Supplementary Table 1.** Scoring Criteria for Coronary Artery Stenosis Severity and Lesion Location Coefficients and Lesion Location Coefficients

| **Degree of Stenosis** | **Score** |
| --- | --- |
| 0-25% | 1 |
| 26-50% | 2 |
| 51-75% | 4 |
| 76-90% | 8 |
| 91-99% | 16 |
| 100% | 32 |
| **Affected Vessel** | **Lesion Site Coefficient** |
| Left Main (LM) | *5 |
| Left Anterior Descending (LAD) | Proximal (*2.5) |
|  | Mid (*1.5) |
|  | Distal (*1) |
| First Diagonal Branch (D1) | *1 |
| Second Diagonal Branch (D2) | *0.5 |
| Third Diagonal Branch (D3) | *0.4 |
| Left Circumflex (LCX) | Proximal (*2.5) |
|  | Mid (*1) |
|  | Distal (*1) |
| Obtuse Marginal Branch (OM) | *1 |
| Posterolateral Branch (PL) | *0.5 |
| Right Coronary Artery (RCA) | Proximal (*1) |
|  | Mid (*1) |
|  | Distal (*1) |
| Posterior Descending Artery (PDA) | *1 |
| Posterolateral Branch (PL) | *0.5 |

**Supplementary Table 2.** Assessment of Multicollinearity: Variance Inflation Factors of Each Predictor

| Variable | VIF |
| --- | --- |
| age | 1.251811 |
| hypertension | 1.125556 |
| DM | 1.059878 |
| smoke | 1.123113 |
| NEU | 2.982772 |
| lnPIV | 3.008386 |
| ALB | 1.066479 |
| LDL.C | 1.239130 |
| AIP | 1.621879 |
| RC | 1.799746 |

In this study, multicollinearity may be introduced as the neutrophil count is a component of the PIV. We chose to retain PIV and exclude neutrophil counts based on considerations of model simplification and improved predictive performance. Although the VIF value did not reach the traditional warning threshold (VIF < 5), this decision effectively avoided information redundancy and improved the model's stability and predictive power.

**Supplementary Table 3.** Quantile regression analysis of the association between Gensini Score and lnPIV and AIP.

| Variable | Quantile | Coefficient | Std. Error | t Value | P |
| --- | --- | --- | --- | --- | --- |
| lnPIV | 0.250 | 1.661 | 1.159 | 1.433 | 0.153 |
|  | 0.500 | 5.899 | 2.923 | 2.018 | 0.045* |
|  | 0.750 | 14.973 | 6.233 | 2.402 | 0.017* |
| AIP | 0.250 | -1.205 | 2.820 | -0.427 | 0.669 |
|  | 0.500 | 12.126 | 7.603 | 1.595 | 0.112 |
|  | 0.750 | 20.969 | 10.326 | 2.031 | 0.043* |

* P value<0.05.
